# Studying human immunodeficiencies in humans: advances in fundamental concepts and therapeutic interventions

**DOI:** 10.12688/f1000research.10594.1

**Published:** 2017-03-24

**Authors:** Helen Su

**Affiliations:** 1 Laboratory of Host Defenses, National Institute of Allergy and Infectious Diseases, National Institutes of Health, Bethesda, MD, USA

**Keywords:** immunodeficiency, immunodeficiencies, immune system, treatments, genomics, model organism

## Abstract

Immunodeficiencies reveal the crucial role of the immune system in defending the body against microbial pathogens. Given advances in genomics and other technologies, this is currently best studied in humans who have inherited monogenic diseases. Such investigations have provided insights into how gene products normally function in the natural environment and have opened the door to new, exciting treatments for these diseases.

## Introduction

The immune system impacts human health in many ways. Some obvious ways are causing autoimmune disease or allergic disease, contributing to immune surveillance against or killing off certain cancers, and determining whether transplanted organs will be accepted or rejected. More recently, the immune system has been recognized as influencing seemingly non-immune conditions. These include the obesity-related metabolic syndrome secondary to chronic low-level inflammation ^1^ as well as possibly even schizophrenia ^2^. However, the immune system mainly evolved to defend against microbial pathogens encountered in early life. Thus, understanding how the human immune system is fundamentally regulated requires understanding how humans respond to infections. This is most directly accomplished by studying immunodeficient patients.

In this brief review, I will define immunodeficiencies and argue that with newer genomics and other technologies, human studies of immunodeficiencies can offer insights equal to or surpassing those previously obtained from mice. In step with the expectation that basic research should lead to improvements in diagnosis and treatment, the ability to study these diseases in humans offers a concrete step towards the development of new immunomodulatory therapies.

## What are immunodeficiencies and why study them?

Inherited human immunodeficiencies are experiments of nature in which gene defects compromise immune function and thereby illuminate how the human immune system works *in vivo*. In the past, immunodeficiencies were recognized by a pattern of recurrent, persistent, or severe infections. Often these included infections caused by unusual microbes: for example, opportunistic infection with *Pneumocystis jirovecii*, which is a hallmark of severe combined immunodeficiency. However, with advances in medical care (antimicrobials, vaccination, etc.) and improvements in hygiene and other societal factors that are keeping patients alive longer, patterns of infection susceptibility in individual patients can be better characterized. This has led to increased recognition of functional redundancy in the immune system that results in some immunodeficiencies presenting with a much narrower infection susceptibility. An example is a group of inherited diseases that primarily present with an isolated susceptibility to Epstein-Barr virus (EBV), manifesting either with a fulminant course including hemophagocytic lymphohistiocytosis or with severe complications such as lymphoma. As this example illustrates, counterintuitively, immunodeficiencies can be accompanied by apparently hyperactive forms of immunodysregulation, including autoimmunity, allergy, lymphoproliferation, and autoinflammation, because of the regulatory complexity of the immune system.

Traditionally, immunodeficiencies have been divided into primary immunodeficiencies, which reflect defects intrinsic to the immune system, or secondary immunodeficiencies, which reflect defects extrinsic to the immune system. Although some secondary immunodeficiencies can be caused by environmental factors such as drugs or irradiation, others can be indirectly caused by non-immune conditions. An example of the latter, primary ciliary dyskinesia, is caused by one of a number of autosomal recessive mutations that affect the structure of cilia on respiratory epithelial cells; by compromising ciliary function, mucus clearance is impaired, leading to bacterial stasis and recurrent sinopulmonary infections.

As shown by the above example, the distinction between primary and secondary immunodeficiencies caused not by environmental factors but by non-immune conditions has increasingly been breaking down with the recognition that non-immune cells can participate in innate immune processes or that global effects more obvious in non-immune cells can occur in parallel in the immune system. The latter is exemplified by many congenital disorders of glycosylation ^3^. Because altered glycosylation patterns can affect protein structure and function, this class of disorders typically causes widespread abnormalities in multiple organ systems, including the nervous system and the immune system. One recently discovered example is caused by autosomal recessive hypomorphic mutations in the phosphoglucomutase-3 ( *PGM3*) gene ^4– 6^. PGM3 encodes an enzyme required for the generation of UDP-Glc-NAc, a building block for *O-* and *N-*linked glycosylation as well as O-Glc-NAc modification involved in signaling. PGM3 deficiency causes a combined immunodeficiency, with neutropenia, autoimmunity, and atopy, although the specific molecules targeted to cause this phenotype have yet to be identified.

Overall, these and other examples have broadened our definition of what constitutes an immunodeficiency and have also rendered fuzzier the distinction between primary and secondary causes of inherited immunodeficiencies due to non-immune conditions.

## Humans: a new, preferred model organism for studying immunity

Immunodeficiencies have often been studied using genetically mutant mice that have arisen spontaneously, after ENU mutagenesis, or after targeted induced mutagenesis (knockouts and knockins). The forward genetics approach has been very successful in uncovering the role of individual gene products in regulating immunity in intact animals. However, laboratory mice are inbred and phenotypes can differ markedly depending upon the genetic background. For example, lpr mutation in mice causes exuberant autoimmunity on the MRL but not the C57BL/6 genetic background. Despite this knowledge, typically only one strain is tested by mouse researchers for any given study, mainly for cost reasons, thus limiting the generalizability of findings to humans, who have diverse genetic backgrounds. Furthermore, it is a concern whether mouse phenotypes tested under highly controlled environments accurately reflect what happens in the natural environment in which people are constantly exposed to different microbes.

For these reasons, humans can be considered a more relevant model for understanding how the immune system is regulated *in vivo*. This trend has been led by advances in genomics. In particular, whole exome sequencing of patients with monogenic disorders has facilitated the unbiased identification of their underlying responsible rare genetic variants. Furthermore, immune cells are easily isolated and tractable to manipulation using genome editing (CRISPR, TALEN), gene silencing (siRNA, shRNA), or lentiviral transduction. In the case of transient progenitors of immune cells or non-immune cells that are not easily accessible, induced pluripotent stem cells from patients can be generated and differentiated into relevant cell types for further study ^7, 8^. These experimental technologies can be used to establish a causal link between the genetic defect and *in vitro* cellular phenotype, even in single-patient studies ^9^. This functional validation of any given candidate disease-causative mutation is an important step required for these types of studies, given that each human is estimated to carry 76 to 190 rare non-synonymous and as many as 20 loss-of-function genetic variants that are potentially deleterious ^10^.

In the past three years, the pace of discovery has accelerated, especially in patients with combined immunodeficiencies or common variable immunodeficiency. Examples of these discoveries are those caused by autosomal or hemizygous recessive mutations in *BCL10* ^11^, *CD70* ^12, 13^, *CTPS1* ^14^, *DOCK2* ^15^, *MSN* ^16^, *NIK* ^17^, *RASGRP1* ^18^, *RLTPR* ^19^, and *TRFC* ^20^; haploinsufficient mutations in *IKAROS* ^21^ or *NFKB1* ^22^; as well as a heterozygous dominant negative mutation in *BCL11B* ^8^. Other examples include humoral immunodeficiency accompanying multi-organ autoimmunity caused by haploinsufficient mutations in *CTLA4* ^23, 24^ or gain-of-function mutations in *STAT3* ^25^; as well as neutrophil dysfunction caused by autosomal recessive mutations in *JAGN1* ^26^ or *WDR1* ^27^. For the most part, these discoveries have dovetailed with findings previously made in mice but have revealed important aspects of the infectious context in which humans manifest disease. For example, human genetic diseases revealed that the CD27–CD70 co-stimulatory pathway has a non-redundant role for EBV control but required identification of patients with corresponding loss-of-function mutations because mice cannot be infected with EBV ^12, 13, 28^. Because of similar experimental limitations, defects in antiviral immunity that confer selective susceptibility to influenza virus or measles/mumps/rubella (caused by autosomal recessive mutations in *IRF7* ^7^ or *IFNAR2* ^29^, respectively) or defects in cytokine production that confer simultaneous susceptibility to Mycobacterial and Candida infections (caused by autosomal recessive mutations in *RORC* ^30^) were first unveiled from studies in humans. In some cases, the work from human patients has also extended work in mice: for example, showing that RASGRP1 regulates cytoskeletal dynamics in immune cells ^18^ or that BCL11B regulates the migration of prethymic hematopoietic progenitors during T cell development ^8^. Finally, with the accumulated discovery of patients having many different gene mutations, larger studies examining the relative roles of these genes involved in signaling for the development of T cell subsets in humans have also been made possible ^31, 32^.

In contrast to monogenic immunodeficiencies, which typically present in early childhood with severe and often lethal outcomes, the genetic contributions to immune responses in older individuals may be less influential, as these would have already been selected out in the general population. Indeed, recent twin studies have established that genetic variations in healthy populations are a minor contributor to immune differences as compared to non-inheritable environmental factors ^33, 34^. This trend was more prominent in older adults, reflecting changes in the adaptive immune system in response to cumulative prior infection history. Systems biology and genomics approaches have made these studies in humans possible, providing new insights into the “healthy” human immune system in the natural environment that could not be obtained from traditional mouse studies. Whether common genetic variants having smaller individual effects together can contribute to milder differences in infection susceptibility in the general population is unknown. If so, increased susceptibility to infection in some situations could be viewed as a complex genetic disease.

## Human studies can drive the discovery of fundamental concepts in basic science

The unbiased discovery approach to human disease genes has led to exciting investigations of the important physiological functions of previously uncharacterized genes that turn out to have major roles in immunity. One recent example is the discovery of autosomal recessive mutations in the tripeptidyl peptidase-2 ( *TPP2*) gene, which causes a combined immunodeficiency, autoimmunity, and neurodevelopmental delay ^35, 36^. Although TPP2 had been previously recognized as an enzyme involved in protein degradation and antigen processing and presentation, the initial genetic discovery and immunophenotyping experiments in the patients led to biochemical studies showing a broader principle governing the immune system (and perhaps analogously in the nervous system): that intracellular amino acid homeostasis is inextricably linked, through aerobic glycolysis, to immune effector functions ^35^.

A second example comes from the clinical observation that patients with autosomal recessive *DOCK8* mutations have a predilection for infections and other pathologies targeting the skin, especially from viruses such as herpes simplex virus that do not cause disease in other tissues ^37^. This observation led to studies that revealed a new concept crucial for anti-viral skin immunity and possibly skin immunity in general: that lymphocytes, through a process controlled by DOCK8, must maintain their shape integrity to survive and exert their functions as they migrate through the highly confined spaces that are unique to skin tissues ^37^.

Finally, a third example of a new discovery coming from studying humans with immunodeficiencies was that of X-linked recessive mutations in *MAGT1*, which causes EBV susceptibility and predisposition to lymphoma by impairing NKG2D expression for anti-viral natural killer (NK) cell activity against EBV-infected cells and immunodeficiency by impairing magnesium-dependent calcium signaling in T cells ^38, 39^. With regard to the latter point, that magnesium can more broadly function as a second messenger for signal transduction was revealed only by delving deeply into understanding these patients. Thus, these three examples demonstrate that studying the immune system through genetic studies in humans yields conceptual scientific advances that go beyond the specific disease being studied.

## Therapeutic interventions as proof-of-principle are becoming a new scientific standard

One of the ways to establish a proposed mechanism of disease involves not only observation but also therapeutic intervention. This is done routinely in mouse studies of the immune system. A good example of this is from a recently reported autoinflammatory disease resulting from autosomal recessive mutations in *OTULIN*, a methionine-1-specific deubiquitinase that normally limits NF-κB activation and hence TNF-associated systemic inflammation ^40^. Using mouse models of the human disease, the authors established the central pathogenic role of dysregulated TNF production when they were able to improve disease by treating with anti-TNF neutralizing antibodies. Importantly, similar effects were obtained when the OTULIN-deficient patients were treated with the TNF antagonist infliximab.

In contrast to hyperactive immune disorders, immunodeficiencies are traditionally cured by hematopoietic stem cell transplantation or gene therapy to restore the missing function, although these modalities can sometimes be associated with mortality and long-term sequelae such as chronic graft-versus-host disease. However, specific knowledge of the pathway affected can also suggest alternative and potentially less risky treatment approaches, even if unlikely to be curative.

A fine example is PASLI (for “p110 delta activating mutation causing senescent T cells, lymphadenopathy, and immunodeficiency” or, alternatively, APDS [for “activated PI3K delta syndrome”]) disease ^41– 44^. This disease is caused by heterozygous gain-of-function mutations in either *PIK3CD* or *PIK3R1*, which encode catalytic or regulatory phosphatidylinositol 3-kinase (PI3K) subunits that are highly expressed in lymphocytes. The mutations hyperactivate mTOR signaling, causing increased senescent CD8 ^+^ T cells at the expense of T cell and B cell memory generation, concurrent lymphoproliferation and hypogammaglobulinemia, and increased infections including poor control of EBV and cytomegalovirus infections. With the knowledge of the signaling pathway affected, the mTOR inhibitor sirolimus has been used in these patients and clinical improvement observed. Fortuitously, targeted therapy using small molecular inhibitors of PI3Kδ are being developed for the treatment of cancers, in which somatic gain-of-function mutations of *PIK3CD* are sometimes found. Thus, PASLI as an orphan disease provides the perfect opportunity to demonstrate precision medicine in action by using the same drugs designed for another purpose. Clinical trials are ongoing to test their efficacy in this disease.

A second example comes from recent work elucidating the molecular mechanism by which LATAIE disease (for “ *LRBA* deficiency with autoantibodies, regulatory T [Treg] cell defects, autoimmune infiltration, and enteropathy”), due to genetic deficiency of the LRBA adaptor protein, causes a mixed picture of autoimmunity and humoral immunodeficiency and responds to CTLA4-Ig (abatacept) treatment ^45^. This therapeutic intervention helped to establish the mechanism by which LRBA deficiency phenocopies CHAI disease (for “ *CTLA4* haploinsufficiency with autoimmune infiltration”) ^23, 24^. Both molecules are interconnected in the same pathway whereby LRBA normally regulates the intracellular vesicle trafficking of, and is required for, cell surface expression of CTLA4. CTLA4 expression, especially on Treg cells, in turn restrains lymphocyte responses and indirectly regulates B cell responses. Furthermore, by defining that LRBA normally helps prevent CTLA4 deposition and degradation in lysosomes, treatment with hydroxychloroquine, a lysosome blocker that is already clinically used for the treatment of lupus, could also be helpful in the treatment of LRBA deficiency.

Together, these two examples illustrate that the unbiased identification of causative gene mutations in human patients leads to knowledge of potential mechanistic targets with immediate clinical implications. Translation of this knowledge has sped up because of the increased development of new biological immunomodulators and repurposing of previously approved medications for new uses. Additionally, knowing what infections result from particular gene mutations can give clues to potential adverse effects in healthy people that might be associated if a new immunomodulatory therapy directed at that molecular target were to be developed.

## Conclusions

Research into immunodeficiencies in humans is now generating exciting new knowledge that is directly relevant for understanding the healthy and diseased immune system and, in some cases, has led to new treatment approaches. Whole exome sequencing technologies are currently driving this trend but have not been able to solve a subset of patients with inherited immunodeficiencies. In this unsolved group of patients, the use of whole genome sequencing in conjunction with transcriptome profiling in relevant cell types could reveal how mutations in non-coding regions such as promoters, enhancers, or other regulatory sequence domains can contribute to human disease. As these could involve temporal as well as tissue-dependent expression during development, functional validation will increasingly require the use of induced pluripotent stem cells or be secondarily complemented by mouse, zebrafish, or other animal models. Finally, because of their immediate relevance to the sick patient, these types of studies are likely to become more closely integrated with clinical interventions that directly demonstrate *in vivo* the central importance of the proposed pathogenic mechanisms.

## References

[1] Odegaard JI Chawla A: Pleiotropic actions of insulin resistance and inflammation in metabolic homeostasis. *Science.* 2013;339(6116):172–7. 10.1126/science.1230721 23307735 PMC3725457

[2] Schizophrenia Working Group of the Psychiatric Genomics Consortium: Biological insights from 108 schizophrenia-associated genetic loci. *Nature.* 2014;511(7510):421–7. 10.1038/nature13595 25056061 PMC4112379

[3] Lyons JJ Milner JD Rosenzweig SD: Glycans Instructing Immunity: The Emerging Role of Altered Glycosylation in Clinical Immunology. *Front Pediatr.* 2015;3:54. 10.3389/fped.2015.00054 26125015 PMC4463932

[4] Zhang Y Yu X Ichikawa M: Autosomal recessive phosphoglucomutase 3 ( *PGM3*) mutations link glycosylation defects to atopy, immune deficiency, autoimmunity, and neurocognitive impairment. *J Allergy Clin Immunol.* 2014;133(5):1400–9,1409.e1–5. 10.1016/j.jaci.2014.02.013 24589341 PMC4016982

[5] Stray-Pedersen A Backe PH Sorte HS: *PGM3* mutations cause a congenital disorder of glycosylation with severe immunodeficiency and skeletal dysplasia. *Am J Hum Genet.* 2014;95(1):96–107. 10.1016/j.ajhg.2014.05.007 24931394 PMC4085583

[6] Sassi A Lazaroski S Wu G: Hypomorphic homozygous mutations in phosphoglucomutase 3 ( *PGM3*) impair immunity and increase serum IgE levels. *J Allergy Clin Immunol.* 2014;133(5):1410–9,1419.e1–13. 10.1016/j.jaci.2014.02.025 24698316 PMC4825677

[7] Ciancanelli MJ Huang SX Luthra P: Infectious disease. Life-threatening influenza and impaired interferon amplification in human IRF7 deficiency. *Science.* 2015;348(6233):448–53. 10.1126/science.aaa1578 25814066 PMC4431581

[8] Punwani D Zhang Y Yu J: Multisystem Anomalies in Severe Combined Immunodeficiency with Mutant *BCL11B*. *N Engl J Med.* 2016;375(22):2165–76. 10.1056/NEJMoa1509164 27959755 PMC5215776

[9] Casanova J Conley ME Seligman SJ: Guidelines for genetic studies in single patients: lessons from primary immunodeficiencies. *J Exp Med.* 2014;211(11):2137–49. 10.1084/jem.20140520 25311508 PMC4203950

[10] 1000 Genomes Project Consortium, Abecasis GR Auton A: An integrated map of genetic variation from 1,092 human genomes. *Nature.* 2012;491(7422):56–65. 10.1038/nature11632 23128226 PMC3498066

[11] Torres JM Martinez-Barricarte R García-Gómez S: Inherited BCL10 deficiency impairs hematopoietic and nonhematopoietic immunity. *J Clin Invest.* 2014;124(12):5239–48. 10.1172/JCI77493 25365219 PMC4348943

[12] Abolhassani H Edwards ES Ikinciogullari A: Combined immunodeficiency and Epstein-Barr virus-induced B cell malignancy in humans with inherited CD70 deficiency. *J Exp Med.* 2017;214(1):91–106. 10.1084/jem.20160849 28011864 PMC5206499

[13] Izawa K Martin E Soudais C: Inherited CD70 deficiency in humans reveals a critical role for the CD70–CD27 pathway in immunity to Epstein-Barr virus infection. *J Exp Med.* 2017;214(1):73–89. 10.1084/jem.20160784 28011863 PMC5206497

[14] Martin E Palmic N Sanquer S: CTP synthase 1 deficiency in humans reveals its central role in lymphocyte proliferation. *Nature.* 2014;510(7504):288–92. 10.1038/nature13386 24870241 PMC6485470

[15] Dobbs K Domínguez Conde C Zhang S: Inherited DOCK2 Deficiency in Patients with Early-Onset Invasive Infections. *N Engl J Med.* 2015;372(25):2409–22. 10.1056/NEJMoa1413462 26083206 PMC4480434

[16] Lagresle-Peyrou C Luce S Ouchani F: X-linked primary immunodeficiency associated with hemizygous mutations in the moesin ( *MSN*) gene. *J Allergy Clin Immunol.* 2016;138(6):1681–1689.e8. 10.1016/j.jaci.2016.04.032 27405666

[17] Willmann KL Klaver S Doğu F: Biallelic loss-of-function mutation in *NIK* causes a primary immunodeficiency with multifaceted aberrant lymphoid immunity. *Nat Commun.* 2014;5:5360. 10.1038/ncomms6360 25406581 PMC4263125

[18] Salzer E Cagdas D Hons M: RASGRP1 deficiency causes immunodeficiency with impaired cytoskeletal dynamics. *Nat Immunol.* 2016;17(12):1352–60. 10.1038/ni.3575 27776107 PMC6400263

[19] Wang Y Ma CS Ling Y: Dual T cell- and B cell-intrinsic deficiency in humans with biallelic *RLTPR* mutations. *J Exp Med.* 2016;213(11):2413–35. 10.1084/jem.20160576 27647349 PMC5068239

[20] Jabara HH Boyden SE Chou J: A missense mutation in *TFRC*, encoding transferrin receptor 1, causes combined immunodeficiency. *Nat Genet.* 2016;48(1):74–8. 10.1038/ng.3465 26642240 PMC4696875

[21] Kuehn HS Boisson B Cunningham-Rundles C: Loss of B Cells in Patients with Heterozygous Mutations in IKAROS. *N Engl J Med.* 2016;374(11):1032–43. 10.1056/NEJMoa1512234 26981933 PMC4836293

[22] Fliegauf M Bryant VL Frede N: Haploinsufficiency of the NF-κB1 Subunit p50 in Common Variable Immunodeficiency. *Am J Hum Genet.* 2015;97(3):389–403. 10.1016/j.ajhg.2015.07.008 26279205 PMC4564940

[23] Kuehn HS Ouyang W Lo B: Immune dysregulation in human subjects with heterozygous germline mutations in *CTLA4*. *Science.* 2014;345(6204):1623–7. 10.1126/science.1255904 25213377 PMC4371526

[24] Schubert D Bode C Kenefeck R: Autosomal dominant immune dysregulation syndrome in humans with *CTLA4* mutations. *Nat Med.* 2014;20(12):1410–6. 10.1038/nm.3746 25329329 PMC4668597

[25] Milner JD Vogel TP Forbes L: Early-onset lymphoproliferation and autoimmunity caused by germline *STAT3* gain-of-function mutations. *Blood.* 2015;125(4):591–9. 10.1182/blood-2014-09-602763 25359994 PMC4304103

[26] Boztug K Järvinen PM Salzer E: JAGN1 deficiency causes aberrant myeloid cell homeostasis and congenital neutropenia. *Nat Genet.* 2014;46(9):1021–7. 10.1038/ng.3069 25129144 PMC4829076

[27] Kuhns DB Fink DL Choi U: Cytoskeletal abnormalities and neutrophil dysfunction in *WDR1* deficiency. *Blood.* 2016;128(17):2135–43. 10.1182/blood-2016-03-706028 27557945 PMC5084607

[28] van Montfrans JM Hoepelman AI Otto S: CD27 deficiency is associated with combined immunodeficiency and persistent symptomatic EBV viremia. *J Allergy Clin Immunol.* 2012;129(3):787–793.e6. 10.1016/j.jaci.2011.11.013 22197273 PMC3294016

[29] Duncan CJ Mohamad SM Young DF: Human IFNAR2 deficiency: Lessons for antiviral immunity. *Sci Transl Med.* 2015;7(307):307ra154. 10.1126/scitranslmed.aac4227 26424569 PMC4926955

[30] Okada S Markle JG Deenick EK: Immunodeficiencies. Impairment of immunity to *Candida* and *Mycobacterium* in humans with bi-allelic *RORC* mutations. *Science.* 2015;349(6248):606–13. 10.1126/science.aaa4282 26160376 PMC4668938

[31] Ma CS Wong N Rao G: Unique and shared signaling pathways cooperate to regulate the differentiation of human CD4 ^+^ T cells into distinct effector subsets. *J Exp Med.* 2016;213(8):1589–608. 10.1084/jem.20151467 27401342 PMC4986526

[32] Ma CS Wong N Rao G: Monogenic mutations differentially affect the quantity and quality of T follicular helper cells in patients with human primary immunodeficiencies. *J Allergy Clin Immunol.* 2015;136(4):993–1006.e1. 10.1016/j.jaci.2015.05.036 26162572 PMC5042203

[33] Roederer M Quaye L Mangino M: The genetic architecture of the human immune system: a bioresource for autoimmunity and disease pathogenesis. *Cell.* 2015;161(2):387–403. 10.1016/j.cell.2015.02.046 25772697 PMC4393780

[34] Brodin P Jojic V Gao T: Variation in the human immune system is largely driven by non-heritable influences. *Cell.* 2015;160(1–2):37–47. 10.1016/j.cell.2014.12.020 25594173 PMC4302727

[35] Lu W Zhang Y McDonald DO: Dual proteolytic pathways govern glycolysis and immune competence. *Cell.* 2014;159(7):1578–90. 10.1016/j.cell.2014.12.001 25525876 PMC4297473

[36] Stepensky P Rensing-Ehl A Gather R: Early-onset Evans syndrome, immunodeficiency, and premature immunosenescence associated with tripeptidyl-peptidase II deficiency. *Blood.* 2015;125(5):753–61. 10.1182/blood-2014-08-593202 25414442 PMC4463807

[37] Zhang Q Dove CG Hor JL: DOCK8 regulates lymphocyte shape integrity for skin antiviral immunity. *J Exp Med.* 2014;211(13):2549–66. 10.1084/jem.20141307 25422492 PMC4267229

[38] Chaigne-Delalande B Li FY O'Connor GM: Mg ^2+^ regulates cytotoxic functions of NK and CD8 T cells in chronic EBV infection through NKG2D. *Science.* 2013;341(6142):186–91. 10.1126/science.1240094 23846901 PMC3894782

[39] Li FY Chaigne-Delalande B Kanellopoulou C: Second messenger role for Mg ^2+^ revealed by human T-cell immunodeficiency. *Nature.* 2011;475(7357):471–6. 10.1038/nature10246 21796205 PMC3159560

[40] Damgaard RB Walker JA Marco-Casanova P: The Deubiquitinase OTULIN Is an Essential Negative Regulator of Inflammation and Autoimmunity. *Cell.* 2016;166(5):1215–1230.e20. 10.1016/j.cell.2016.07.019 27523608 PMC5002269

[41] Lucas CL Kuehn HS Zhao F: Dominant-activating germline mutations in the gene encoding the PI(3)K catalytic subunit p110δ result in T cell senescence and human immunodeficiency. *Nat Immunol.* 2014;15(1):88–97. 10.1038/ni.2771 24165795 PMC4209962

[42] Angulo I Vadas O Garçon F: Phosphoinositide 3-kinase δ gene mutation predisposes to respiratory infection and airway damage. *Science.* 2013;342(6160):866–71. 10.1126/science.1243292 24136356 PMC3930011

[43] Lucas CL Zhang Y Venida A: Heterozygous splice mutation in *PIK3R1* causes human immunodeficiency with lymphoproliferation due to dominant activation of PI3K. *J Exp Med.* 2014;211(13):2537–47. 10.1084/jem.20141759 25488983 PMC4267241

[44] Deau MC Heurtier L Frange P: A human immunodeficiency caused by mutations in the *PIK3R1* gene. *J Clin Invest.* 2014;124(9):3923–8. 10.1172/JCI75746 25133428 PMC4153704

[45] Lo B Zhang K Lu W: AUTOIMMUNE DISEASE. Patients with LRBA deficiency show CTLA4 loss and immune dysregulation responsive to abatacept therapy. *Science.* 2015;349(6246):436–40. 10.1126/science.aaa1663 26206937

